# Moral foundations are better predictors of belief in COVID-19 conspiracy theories than the Big Five personality traits

**DOI:** 10.3389/fpsyg.2023.1201695

**Published:** 2023-08-24

**Authors:** Pegah Nejat, Ali Heirani-Tabas, Mohammad Mahdi Nazarpour

**Affiliations:** Department of Psychology, Faculty of Education and Psychology, Shahid Beheshti University, Tehran, Iran

**Keywords:** Moral Foundations Theory (MFT), Liberty, conspiracy beliefs, COVID-19 pandemic, Big Five, personality

## Abstract

Upon the sudden outbreak of COVID-19 pandemic, various conspiracy theories regarding the virus proliferated in the social media. This study focused on the sociodemographic, personality, and moral predictors of these beliefs. More specifically, we asked whether moral values predict belief in COVID-19 conspiracy theories over and above sociodemographic variables and the Big Five personality traits. According to Moral Foundations Theory, five cross-cultural moral foundations are more broadly categorized under individualizing (Care & Fairness) and binding (Loyalty, Authority, & Sanctity) foundations. A sixth moral foundation was Liberty which we included along with binding and individualizing foundations. Participants were 227 Iranians (mean age = 31.43, SD = 12.61, 75.3% female) who responded to Moral Foundations Questionnaire and Liberty items, a range of COVID-19 conspiracy beliefs, and the 10-Item Personality Measure of the Big Five. Among demographic variables, religiosity and socioeconomic status were the strongest determinants of conspiracy beliefs regarding the origin of Coronavirus. Among the Big Five, only extraversion predicted these beliefs in a positive direction. Moral foundations, most notably Authority and Sanctity, showed incremental predictive power over both demographic variables and the Big Five personality traits. Findings are discussed in light of the role of social media in dissemination of conspiracy beliefs regarding the pandemic. They point to the more relevance of moral foundations, particularly binding foundations, than the Big Five in the context of pandemic-related conspiracy beliefs, and add to the literature on the unique contribution of moral foundations to socio-political attitudes across cultures.

## 1. Introduction

In December 2019, the world was shocked by the news on emergence of a cold-like disease in Wuhan, China, and the subsequent lockdown due to that. The World Health Organization published the first announcement about the virus, named as COVID-19, on January 5, 2020, and declared it a pandemic on March 11 (World Health Organization, 2020). The first deaths in Iran were reported on Feb 19, followed by a rapid spread of the disease, such that in April, 2020 it ranked sixth among world countries in total COVID-19 deaths (Nikpouraghdam et al., 2020).

The pandemic led to a substantial increase in information seeking concerning the disease (e.g., Casero-Ripollés, 2020), with certain beliefs spreading through the social media, and giving rise to an “infodemic” in parallel to the pandemic (Infodemic, 2020). Examples include a China-produced bioweapon and links to Internet 5G or Bill Gates (Shahsavari et al., 2020). The Infodemic Risk Index, a measure of exposure to unreliable news via Twitter, was found to be particularly high in Iran (Gallotti et al., 2020). Later, beliefs similar to the ones concerning origin of the virus targeted vaccination. In September 2021, skepticism about the vaccine was the first among the two main reasons underlying lack of interest in getting vaccinated among Iranians (Iranian Students Poll Agency, 2021). The present study seeks to shed light on the sociodemographic, personality, and moral underpinnings of these pandemic-related beliefs in Iran.

Beliefs similar to the ones mentioned in relation to COVID-19 have existed in the literature under the title of conspiracy theories (Goertzel, 1994; Zonis and Joseph, 1994; Aaronovitch, 2010). Generally defined as a set of beliefs providing explanations about ultimate causes of a significant social event by attributing them to mostly powerful agents (Dentith and Orr, 2017), conspiracy theories have long constituted part of the human history, and are particularly likely to show up in response to social crises (Van Prooijen and Douglas, 2017). The sudden outbreak of COVID-19 with its noticeable impact on social life worldwide represented one such event. COVID conspiracy theories entail various beliefs regarding the artificiality or exploitation of the virus (Freeman et al., 2020;Imhoff and Lamberty, 2020; Karić and Međedović, 2021), and centering around malevolent groups pursuing specific objectives, or governments taking advantage of it to advance anti-democratic goals (Oleksy et al., 2021). The beliefs have been linked to low education, young age and low income (Romer and Jamieson, 2020), and lead to more spread of the disease through less commitment to preventive behaviors (Imhoff and Lamberty, 2020; Karić and Međedović, 2021). As such, identifying determinants of these beliefs bears significant practical implications.

As a culture-sensitive theory in moral psychology, Moral Foundations Theory (Haidt and Joseph, 2004; Graham et al., 2013) distinguished between five flavors of moral intuition underpinning moral judgment. Moral foundations include Care/Harm, Fairness/Cheating, Loyalty/Betrayal, Authority/Subversion, and Sanctity/Degradation, more broadly categorized under individualizing (Care and Fairness) and binding (Loyalty, Authority, and Sanctity) foundations (Graham et al., 2011; Nejat and Hatami, 2019). A sixth foundation, namely Liberty/Oppression, was proposed later by Iyer et al. (2012). We also included this rather understudied moral foundation in this study along with individualizing and binding foundations.

The focus on ingroup survival underlying binding foundations (Graham et al., 2011), is congruent with the “us vs. them” mentality inherent in most conspiracy beliefs (Van Prooijen and Van Lange, 2014). Further, conspiracy-evoking events are usually considered as a threat to the society (Leone et al., 2019), thus tapping on the motive to conserve the group. Consistently, binding and individualizing foundations had positive and negative relations with conspiracy beliefs, respectively, with binding foundations more strongly relating to these beliefs (Leone et al., 2019). Similarly, more concern with moral purity, as reflected in sexual and dietary prohibitions, predicted higher endorsement of COVID conspiracy beliefs in Finland (Pivetti et al., 2021), while Nestik and Deyneka (2020) found positive associations between COVID conspiracy beliefs and endorsement of Loyalty and Authority in Russia. Finally, endorsement of Care and Fairness predicted higher trust in science and government (Pagliaro et al., 2021). Considering the skepticism of conspiracy theories toward official accounts of events (Connolly et al., 2019), including those originating from scientists or governments, as well as empirical findings suggesting a negative link between trust in science/government and COVID conspiracy beliefs (Freeman et al., 2020; Kim and Kim, 2021; Vranic et al., 2022), more reliance on Care and Fairness may be argued to counteract conspiratorial thinking through higher trust in science/government. Accordingly, we anticipate a positive relation between binding foundations and COVID conspiracy beliefs, and a negative association between individualizing foundations and these beliefs, with a more salient role for binding foundations.

Empirical evidence supports incremental predictive power of moral foundations beyond a number of variables, e.g., beyond Schwartz values in predicting attitude toward social groups (Graham et al., 2011), beyond core motives in predicting general political orientation (Yilmaz and Saribay, 2019), and beyond personal values of self-enhancement/self-transcendence and openness to change/conservation in predicting behavior and attitudes (Feldman, 2021). The current study aims to contribute to this literature by examining incremental predictive power of moral foundations beyond the Big Five of personality (McCrae and Costa, 1999), namely extraversion, agreeableness, conscientiousness, neuroticism (also called emotional stability, its reverse), and openness to experience, in determining conspiracy beliefs surrounding COVID-19.

The Big Five traits may be argued to contribute in distinct ways to the motives (Douglas et al., 2019) underlying conspiracy beliefs (hereafter referred to as our ‘motives account’). More specifically, given that neuroticism entails heightened reaction to stress, it may thus strengthen the existential (security and control) motive, functioning to drive conspiracy beliefs. Conversely, the trust and optimism underpinning agreeableness would possibly reduce the salience of this motive, resulting in less proneness to conspiracy theories. Extraverts’ concern with social relationships may contribute to the importance of the social (maintaining positive image of self and ingroup) motive to them, rendering them prone to conspiracy beliefs. Given the link between conspiracy beliefs and the need for cognitive closure (e.g., Marchlewska et al., 2018), indicative of the epistemic (understanding) motive (Douglas et al., 2019), openness to experience, an opposite of tendency for closure, can be expected to predict less vulnerability to conspiracies. Finally, despite the high-conscientious’ interest in order and accuracy, their lack of impulsivity would lead them to seek more rational means than conspiracy beliefs in order to satisfy epistemic (understanding) needs. In sum, we expect neuroticism and extraversion to relate positively, but agreeableness, conscientiousness, and openness to relate negatively to conspiracy beliefs.

Empirical evidence suggests contribution of the Big Five to COVID-related stress and anxiety (Bellingtier et al., 2021; Nikčević et al., 2021; Pérez-Mengual et al., 2021; Zacher and Rudolph, 2021; Lassen et al., 2022). Given the positive link between anxiety/stress and COVID conspiracy beliefs (Sallam et al., 2020; Hartman et al., 2021; Barahmand et al., 2022; Pfeffer et al., 2022; Šrol et al., 2022; Liekefett et al., 2023), the Big Five may also serve as predictor of COVID conspiracy beliefs. Common predictors among the above-cited studies are neuroticism and extraversion. They converged on the higher vulnerability of high-neurotic individuals to COVID anxiety/stress, while partly disagreeing on the role of extraversion. Whereas some found extraversion a protective factor against COVID-related worry/anxiety (Nikčević et al., 2021; Pérez-Mengual et al., 2021; Lassen et al., 2022), others reported opposing relations for different timespans (Zacher and Rudolph, 2021) or facets (Bellingtier et al., 2021). Taken together, relying on anxiety/stress as the mechanism underlying the relationship between the Big Five and conspiracy beliefs (hereafter referred to as our ‘stress account’), whereas neuroticism may be expected to positively predict COVID conspiracy beliefs, there is more ambiguity regarding the role of extraversion.

The literature on the association between conspiracy beliefs and the Big Five also contains inconsistencies. Whereas some found positive relations between conspiracy beliefs and openness (Swami and Furnham, 2012; Swami et al., 2013; Charlton, 2014), agreeableness (Swami et al., 2010; Orosz et al., 2016), and conscientiousness (Swami and Furnham, 2012; Charlton, 2014; Arnulf et al., 2022), others reported negative relations between these beliefs and openness (Orosz et al., 2016), agreeableness (Swami and Furnham, 2012; Charlton, 2014; Bowes et al., 2021; Arnulf et al., 2022), and conscientiousness (Brotherton et al., 2013; Bowes et al., 2021). However, the reported significant relations between conspiracy beliefs and neuroticism or extraversion all converged on positive associations with both neuroticism (Swami and Furnham, 2012; Swami et al., 2013; Charlton, 2014; Hollander, 2018; Heiss et al., 2021) and extraversion (Heiss et al., 2021; Arnulf et al., 2022). Nevertheless, the majority of the cited studies addressed non-COVID conspiracy theories. Given the distinct nature of the recent pandemic, such as reduced social contact and individuals’ over-reliance on social media as a result of that, different findings may emerge regarding the conspiracy beliefs relating to it. In response to the presented gaps with respect to the Big Five, as well as the small number of studies examining the link between moral foundations and COVID conspiracy beliefs, this study aimed to investigate the role of the Big Five traits and moral foundations as determinants of COVID-19 conspiracy beliefs in an understudied non-Western context, with expectation of more salient roles for binding foundations, neuroticism, and extraversion.

## 2. Methods

### 2.1. Participants

Participants were 253 Iranians who were recruited through social media, and responded to the survey via an Iranian survey-hosting website during April–June 2021. 26 participants were excluded due to incorrect responses to the attention check, leaving a final sample of *N* = 227 (mean age = 31.43, *SD* = 12.61, 75.3% female) ranging in age from 17 to 71 years. The majority of participants already had or were pursuing a Bachelor’s degree (61.7%), followed by Master’s or M.D. (17.7%), primary or high school (15.4%), and Ph.D. (5.3%). This study received approval of the departmental review board, and informed consent was obtained from all participants.

### 2.2. Materials

#### 2.2.1. Moral foundations and Liberty

The Persian translation (Nejat and Hatami, 2019) of the 20-item Moral Foundations Questionnaire (MFQ-20; Graham et al., 2011) was used to assess endorsement of Care, Fairness, Loyalty, Authority, and Sanctity. MFQ consists of relevance and judgment sections, and each moral foundation is represented by two items in each section. Responses ranged from 0 (Not at all important) to 5 (Extremely important), and from 0 (Strongly disagree) to 5 (Strongly agree) for relevance and judgment, respectively. Omega coefficients were Care 0.37, Fairness 0.65, Loyalty 0.64, Authority 0.70, and Sanctity 0.68. Due to the Care’s low internal consistency, Care and Fairness were aggregated to form individualizing foundations (*ω* = 0.68) whereas Loyalty, Authority, and Sanctity constituted the binding foundations (*ω* = 0.84). The Persian translation (Nejat and Hatami, 2019) of seven out of the nine items proposed by Iyer et al. (2012) was used to assess Liberty, consisting of two relevance and five judgment items (*ω* = 0.66).

#### 2.2.2. Belief in COVID-19 conspiracy theories

A pool of 34 items was accumulated from a set of relevant studies (Freeman et al., 2020; Imhoff and Lamberty, 2020; Olatunji et al., 2020; Eberl et al., 2021; Karić and Međedović, 2021) with responses ranging from 1 (Strongly disagree) to 5 (Strongly agree). To arrive at the final measure, we gathered the whole set of items through the cited studies, identified redundant ones across scales, and excluded the ones decided as the most irrelevant to the context of our culture and country based on judgment of the principal investigator and two lab assistants. The final set of items were categorized as the following based on their content: Lockdown (three items, *α* = 0.71), Vaccination (three items, *α* = 0.83), Virus origin (21 items, *α* = 0.93), Exploitation of COVID for other purposes (three items, *α* = 0.78), and News accuracy (four items, *α* = 0.62). Sample items include “The intention of lockdown is to force people to rely on big corporations rather than local businesses” (Lockdown), “The coronavirus vaccine contains microchips to control people” (Vaccination), “The spread of the virus is a deliberate attempt to reduce the size of the global population” (Virus origin), “Coronavirus is a news only to divert attention from more important things” (Exploitation), and “The mainstream media is deliberately feeding us misinformation about the virus and lockdown” (News accuracy). Six reverse-scored items referred to formal facts regarding COVID, four of them in the virus origin, e.g., “The virus is naturally occurring,” and two in news accuracy. The full set of items is presented in Supplementary materials. Cronbach alpha for the whole set of items was 0.95. Confirmatory factor analysis of a five-correlated-factor model yielded *CFI* = 0.77, *RMSEA* = 0.097, and *CMIN*/*df* = 3.11, with regression weights all significant, *p*s < 0.05.

#### 2.2.3. The Big Five

We used the Persian translation (Azkhosh et al., 2019) of the 10-Item Personality Inventory (TIPI; Gosling et al., 2003). Each dimension of the Big Five was represented by two items with responses ranging from 1 (Strongly disagree) to 5 (Strongly agree). Cronbach alphas were Extraversion 0.70, Agreeableness 0.34, Conscientiousness 0.56, Emotional Stability 0.37, and Openness to Experience 0.43. Studies have reported similarly low alphas for this scale, e.g., 0.40–0.73 (Gosling et al., 2003), 0.40–0.69 (Azkhosh et al., 2019), and 0.38–0.61 (Romero et al., 2012). Gosling et al. (2003) justified the use of this scale, despite mediocre internal consistency, by prioritizing content validity through minimum number of items, and satisfactory test–retest reliability and validity.

#### 2.2.4. Demographics

Participants reported their age, gender, political orientation (from 1 = “Very principlist” to 5 = “Very reformist”), religiosity (from 1 = “Not at all or very little” to 5 = “Very much”), and socioeconomic status (SES; from 1 = “Very lower than average” to 5 = “Very higher than average”).

## 3. Results

### 3.1. Correlations

Individualizing moral foundations were inversely correlated only with beliefs on news accuracy, whereas binding moral foundations were positively correlated with all conspiracy belief categories except news accuracy. Liberty had negative but weak correlations with all belief categories (Table 1). Extraversion was positively correlated only with virus origin beliefs, while the remaining four Big Five dimensions were not significantly related to any of the belief categories. Regarding sociodemographics, political orientation was negatively correlated with vaccination, origin, and exploitation beliefs, while religiosity was positively related to all belief categories, and SES was not significantly correlated with any of the belief categories, but marginally related to lockdown, *p* = 0.054, vaccination, *p* = 0.054, and exploitation, *p* = 0.061. Age was not significantly related to any of the belief categories. The few significant correlations between moral foundations and the Big Five were all weak in size, *rs* < 0.24.

**Table 1:** Descriptive statistics and zero-order correlations between COVID conspiracy beliefs, moral foundations, the Big Five, and sociodemographic variables.

	1	2	3	4	5	6	7	8	9	10	11	12	13	14	15	16	17	*M*	SD
1. Lockdown	–	0.66^***^	0.63^***^	0.71^***^	0.51^***^	−0.04	0.30^***^	−0.14^*^	0.06	0.03	−0.01	−0.04	0.02	−0.08	0.20^**^	−0.13	0.07	2.15	0.91
2. Vaccination		–	0.73^***^	0.81^***^	0.56^***^	−0.09	0.28^***^	−0.19^**^	0.06	0.07	0.01	0.07	0.00	−0.13^*^	0.32^***^	−0.13	0.08	2.12	0.98
3. Virus origin			–	0.77^***^	0.48^***^	0.03	0.37^***^	−0.19^**^	0.18^**^	0.07	0.04	−0.01	0.01	−0.20^**^	0.37^***^	−0.06	0.10	2.60	0.79
4. Exploitation				–	0.58^***^	−0.08	0.30^***^	−0.22^***^	0.11	0.08	−0.01	−0.02	−0.03	−0.14^*^	0.29^***^	−0.12	0.11	2.25	0.98
5. News accuracy					–	−0.28^***^	0.02	−0.21^**^	−0.02	0.03	0.01	0.09	0.02	0.04	0.18^**^	−0.10	−0.02	1.88	0.68
6. Individualizing foundations						–	0.36^**^	0.28^***^	0.07	0.12	0.23^***^	−0.06	0.06	−0.04	−0.06	0.10	0.01	4.00	0.61
7. Binding foundations							–	−0.06	0.11	0.10	0.16^*^	0.01	−0.16^*^	−0.21^**^	0.48^***^	0.09	0.32^***^	2.99	0.85
8. Liberty								–	−0.01	0.09	0.05	−0.00	0.14^*^	0.16^*^	−0.27^***^	−0.03	−0.06	3.75	0.69
9. Extraversion									–	−0.00	−0.06	−0.08	0.13^*^	−0.01	0.09	0.09	0.13	3.23	1.10
10. Agreeableness										–	0.34^***^	0.21^**^	0.21^**^	−0.01	0.01	−0.02	0.12	3.88	0.84
11.Conscientiousness											–	0.09	0.16^*^	−0.04	0.04	0.08	0.12	3.78	0.95
12. Emotional stability												–	0.14^*^	0.04	0.13^*^	0.05	0.02	2.98	0.94
13. Openness to experience													–	0.01	−0.14^*^	−0.02	−0.16^*^	3.74	0.83
14. Political orientation														–	−0.20^**^	0.04	−0.04	3.14	0.61
15. Religiosity															–	0.15^*^	0.11	2.81	0.99
16. SES																–	−0.05	3.16	0.74
17. Age																	–	31.43	12.61

^*^p < 0.05, ^**^p < 0.01, ^***^p < 0.001.

### 3.2. Incremental predictive power of moral foundations

Except the weak correlations between extraversion and total COVID conspiracy beliefs, *r* = 0.15, *p* = 0.026, and between extraversion and origin conspiracy beliefs (Table 1), all other correlations between the Big Five and total conspiracy beliefs, rs < 0.08, *ps* > 0.27, or the belief categories, *p*s > 0.095, were non-significant. Given that the strongest correlation involved origin conspiracy beliefs, we used this category of beliefs as the criterion variable. We conducted a hierarchical regression analysis entering demographic variables in the first step, followed by the Big Five and moral foundations in the second and third steps, respectively (Table 2). We entered binding foundations as separate moral foundations in this analysis in order to gain a more detailed picture of their relationship with conspiracy beliefs. As revealed, highly religious individuals endorsed more origin conspiracy beliefs. Among personality factors, only extraversion, with a positive coefficient, was a significant predictor of origin conspiracy beliefs. The change in explained variance due to personality factors was not significant, *F*(5, 216) = 1.69, *p* = 0.138. Among moral foundations, Sanctity and Authority were significant positive predictors. The significant change in R square in step 3, *F*(5, 211) = 4.78, *p* < 0.001, indicated the incremental predictive power of moral foundations beyond both demographic variables and the Big Five. The final step also included socioeconomic status (SES) as a significant negative predictor.

**Table 2:** Hierarchical regression analysis with sociodemographic variables, the Big Five, and moral foundations as predictors of virus origin conspiracy beliefs.

Category	Predictor	Step 1		Step 2		Step 3	
		*β*	SE	*β*	SE	*β*	SE
Socio-demographics	Age	0.05	0.00	0.03	0.00	−0.04	0.00
	Gender	−0.04	0.11	−0.02	0.12	−0.01	0.11
	Religiosity	0.35^***^	0.05	0.35^***^	0.05	0.21^**^	0.06
	SES	−0.11^†^	0.07	−0.12^†^	0.07	−0.12^*^	0.06
	Political orientation	−0.12^†^	0.08	−0.12^†^	0.08	−0.05	0.08
The Big Five	Emotional stability			−0.05	0.05	−0.03	0.05
	Openness to experience			0.04	0.06	0.07	0.06
	Conscientiousness			0.01	0.06	−0.01	0.05
	Extraversion			0.15^*^	0.05	0.14^*^	0.04
	Agreeableness			0.05	0.06	0.05	0.06
Moral foundations	Individualizing					−0.00	0.09
	Sanctity					0.18^*^	0.07
	Authority					0.28^***^	0.06
	Loyalty					−0.12	0.07
	Liberty					−0.08	0.07
*R^2^* (SE)		0.17^***^ (0.73)		0.20^***^ (0.72)		0.28^***^ (0.69)	
∆*R^2^*		0.17^***^		0.03		0.08^***^	

^†^*p* < 0.10, ^*^p < 0.05, ^**^p < 0.01, ^***^p < 0.001; *β* is standardized regression coefficient.

A similar hierarchical regression analysis with total COVID conspiracy beliefs as the criterion variable yielded a non-significant, *F*(5, 216) = 1.10, *p* = 0.359, and significant, *F*(5, 211) = 5.08, *p* < 0.001, changes in R square by adding the Big Five and moral foundations to the model, respectively. Across the three steps, religiosity, 0.19 < *β*s < 0.36, *p*s < 0.05, and SES, −0.16 < *β*s < −0.14, *p*s < 0.05, were positive and negative predictors of conspiracy beliefs, respectively. Extraversion was a marginally significant positive predictor in both the second, *β* = 0.12, *SE* = 0.04, *p* = 0.072, and the third, *β* = 0.11, *SE* = 0.04, *p* = 0.088, steps. Among moral foundations, only Sanctity, *β* = 0.20, *SE* = 0.06, *p* = 0.020, and Authority, *β* = 0.26, *SE* = 0.06, *p* = 0.002, turned out as significant predictors of total COVID conspiracy beliefs, both in the positive direction. All other coefficients were non-significant, |*β*|s < 0.11, *p*s > 0.10.

## 4. Discussion

As expected, binding foundations tended to have stronger relationships with COVID conspiracy beliefs than both individualizing foundations and Liberty. Further, moral foundations explained both total COVID conspiracy beliefs, and more specifically, conspiracy beliefs regarding the virus origin, beyond sociodemographic characteristics and the Big Five, thereby adding to the literature on incremental predictive power of moral foundations, also complementing it by including the understudied moral foundation of Liberty. More specifically, higher reliance on Authority and Sanctity predicted more endorsement of COVID conspiracy beliefs, a finding consistent with current evidence on the association between binding foundations and conspiracy beliefs (Leone et al., 2019; Nestik and Deyneka, 2020; Pivetti et al., 2021). Although we also expected Loyalty (Nestik and Deyneka, 2020) and individualizing foundations (Leone et al., 2019; Pagliaro et al., 2021) to predict conspiracy beliefs, our findings revealed a more salient role for Authority and Sanctity. Individuals relying more heavily on Authority to whom deference to authority figures is essential, may be particularly willing to gain control amid the pandemic by securing a role for the high-powered, as generally indicated by conspiracy beliefs (Dentith and Orr, 2017). Moreover, individuals high in Sanctity who are particularly tuned at purity concerns (Graham et al., 2013), may be subscribing to conspiracy beliefs in order to dismiss the prospect of contamination by either the virus itself or the formal measures associated with that, e.g., use of chemical drugs or vaccines.

The Big Five traits did not emerge as strong predictors of conspiracy beliefs, with only more extraverted individuals reporting higher endorsement of beliefs regarding the COVID origin, as expected based on both our proposed relation between extraversion and the social motive, and prior empirical evidence on the association between extraversion and conspiracy beliefs (e.g., Heiss et al., 2021; Arnulf et al., 2022). This finding is also in line with the large array of studies suggesting a more salient role for extraversion in prediction of social media use compared to the remaining four Big Five dimensions. Extraversion has been the most robust correlate of social media use followed by openness to experience and neuroticism (Correa et al., 2010; Wang et al., 2012; Liu and Campbell, 2017; Bowden-Green et al., 2020; Marengo et al., 2020), with extraverts spending more time online and having larger social networks. This can lead to their higher exposure to COVID conspiracy beliefs, especially in the context of Iran’s high Infodemic Risk Index (Gallotti et al., 2020), and result in higher endorsement of these beliefs as a consequence.

Although openness to experience has also been found to predict higher social media use (Correa et al., 2010; Liu and Campbell, 2017), its positive effect on conspiracy beliefs might have been counteracted by its negative impact on conspiracy beliefs through lower epistemic motive as suggested by our motives account. We also anticipated emotional stability to negatively predict endorsement of COVID conspiracy beliefs based on both our motives account and stress account, and similar primarily non-COVID empirical findings. Consistently, high-neurotics’ relatively high use of social media (Correa et al., 2010; Liu and Campbell, 2017; Marengo et al., 2020) indicates that they should encounter conspiracy beliefs rather frequently. The lack of support for the relationship between this dimension and conspiracy beliefs in this study may be due to high-neurotics’ tendency to avoid exposure to social media during the pandemic as a coping strategy, given the distress evoked by their content, resulting in high-neurotics’ less exposure to conspiracy beliefs.

Despite our motives-based predictions for negative relations between both agreeableness and conscientiousness and conspiracy beliefs, we found no evidence of these links, in line with the mixed or weak evidence associated with them. Some prior studies have likewise obtained less than robust associations with conspiracy beliefs across all (Brotherton et al., 2013; Bruder et al., 2013) or a selective set (agreeableness and openness; Leiser et al., 2017) of the Big Five, just as some meta-analyses failed to find strong evidence in support of this relationship (Goreis and Voracek, 2019; Stasielowicz, 2022), despite heterogeneity among studies (Stasielowicz, 2022). Three explanations may account for this finding. First, more specific personality traits such as narcissism (Gligorić et al., 2021; Sternisko et al., 2023) or schizotypal tendencies (Swami et al., 2013; Stasielowicz, 2022) may be better determinants of conspiracy beliefs than the generic Big Five. Second, conspiracy beliefs might be more substantially influenced by situational conditions, and (sub-)cultural or religious beliefs rather than personality dispositions. Third, the less than robust association between the Big Five and conspiracy beliefs may be due to the simultaneous activation of multiple mechanisms with diverging effects, as suggested for openness to experience.

Among demographics, only religiosity and SES predicted COVID conspiracy beliefs both in total and with respect to virus origin, with more religious or lower-SES individuals endorsing more of these beliefs. This resonates with prior cross-cultural findings on SES (e.g., Georgiou et al., 2020; Romer and Jamieson, 2020; Tonković et al., 2021; Hettich et al., 2022), and religiosity (e.g., Alper et al., 2021; Dyrendal and Hestad, 2021; Stasielowicz, 2022; Frenken et al., 2023). However, findings on gender and age are more inconsistent. Whereas some obtained no gender differences (Freeman et al., 2020; Dyrendal and Hestad, 2021; Tonković et al., 2021), others found women (Erceg et al., 2020; Alper et al., 2021; Vranic et al., 2022) or men (Cassese et al., 2020; Hettich et al., 2022) higher in these beliefs. Likewise, whereas some have concluded that age relates to conspiracy beliefs (Freeman et al., 2020; Romer and Jamieson, 2020; Hettich et al., 2022), others (e.g., Dyrendal and Hestad, 2021; Tonković et al., 2021; Vranic et al., 2022) found age unrelated to these beliefs, as the current study.

### 4.1. Limitations and future directions

One limitation of this study is its reliance on conspiracy items from non-Iranian studies. Although cultures may be expected to differ with respect to conspiracy theories, given the lack of qualitative studies specific to our culture, the decision may be justified through the inclusion of studies from various countries, and the omission of the conspiracy beliefs that we judged as irrelevant to our culture. Future researchers are recommended to explore determinants of more culturally-dependent conspiracy beliefs in addition to the universal ones.

## 5. Conclusion

This study examined the sociodemographic, personality, and moral determinants of COVID-19 conspiracy beliefs in the context of the Iranian culture. Findings suggest the more relevance of moral values, most notably binding foundations of Authority and Sanctity, than the Big Five traits to the way individuals respond to a pandemic in terms of conspiratorial thinking, adding to the literature on the unique contribution of moral foundations to socio-political attitudes across cultures.

## Data availability statement

The raw data supporting the conclusions of this article will be made available by the authors, without undue reservation.

## Ethics statement

The studies involving humans were approved by Departmental Board at Faculty of Education and Psychology, Shahid Beheshti University. The studies were conducted in accordance with the local legislation and institutional requirements. The participants provided their written informed consent to participate in this study.

## Author contributions

PN: conceptualized, designed the study, supervised the project, and wrote the first draft of the manuscript. PN and AH-T: performed the statistical analyses. AH-T and MN: contributed to literature review and wrote sections of the manuscript. All authors contributed to manuscript revision, read, and approved the submitted version.

## Conflict of interest

The authors declare that the research was conducted in the absence of any commercial or financial relationships that could be construed as a potential conflict of interest.

## Publisher’s note

All claims expressed in this article are solely those of the authors and do not necessarily represent those of their affiliated organizations, or those of the publisher, the editors and the reviewers. Any product that may be evaluated in this article, or claim that may be made by its manufacturer, is not guaranteed or endorsed by the publisher.

## References

[ref1] Aaronovitch D. (2010). Voodoo histories: The role of the conspiracy theory in shaping modern history. New York, NY: Riverhead Books.

[ref2] Alper S. Bayrak F. Yilmaz O. (2021). Psychological correlates of COVID-19 conspiracy beliefs and preventive measures: evidence from Turkey. Curr. Psychol. 40, 5708–5717. doi: 10.1007/s12144-020-00903-0, PMID: 32837129 PMC7324005

[ref3] Arnulf J. K. Robinson C. Furnham A. (2022). Dispositional and ideological factor correlate of conspiracy thinking and beliefs. PLoS One 17:e0273763. doi: 10.1371/journal.pone.0273763, PMID: 36288289 PMC9604007

[ref4] Azkhosh M. Sahaf R. Rostami M. Ahmadi A. (2019). Reliability and validity of the 10-item personality inventory among older Iranians. Psychol. Russ. 12, 28–38. doi: 10.11621/pir.2019.0303

[ref5] Barahmand U. Mohamadpour S. Sheikh Ahmad R. H. (2022). COVID-19 related stresses, conspiracy beliefs, uncertainty, and non-adherence to safety guidelines. Int. J. Psychol. Res. 15, 22–33. doi: 10.21500/20112084.5367, PMID: 37274517 PMC10233957

[ref6] Bellingtier J. A. Mund M. Wrzus C. (2021). The role of extraversion and neuroticism for experiencing stress during the third wave of the COVID-19 pandemic. Curr. Psychol. 42, 12202–12212. doi: 10.1007/s12144-021-02600-y, PMID: 34903948 PMC8655714

[ref7] Bowden-Green T. Hinds J. Joinson A. (2020). How is extraversion related to social media use? A literature review. Personal. Individ. Differ. 164:110040. doi: 10.1016/j.paid.2020.110040

[ref8] Bowes S. M. Costello T. H. Ma W. Lilienfeld S. O. (2021). Looking under the tinfoil hat: clarifying the personological and psychopathological correlates of conspiracy beliefs. J. Pers. 89, 422–436. doi: 10.1111/jopy.12588, PMID: 32852781

[ref9] Brotherton R. French C. C. Pickering A. D. (2013). Measuring belief in conspiracy theories: the generic conspiracist beliefs scale. Front. Psychol. 4:279. doi: 10.3389/fpsyg.2013.00279, PMID: 23734136 PMC3659314

[ref10] Bruder M. Haffke P. Neave N. Nouripanah N. Imhoff R. (2013). Measuring individual differences in generic beliefs in conspiracy theories across cultures: conspiracy mentality questionnaire. Front. Psychol. 4:225. doi: 10.3389/fpsyg.2013.00225, PMID: 23641227 PMC3639408

[ref11] Casero-Ripollés A. (2020). Impact of COVID-19 on the media system. Communicative and democratic consequences of news consumption during the outbreak. Casero-Ripollés, Andreu (2020). “Impact of COVID-19 on the media system. Communicative and democratic consequences of news consumption during the outbreak”. El profesional de la información 29:e290223. doi: 10.3145/epi.2020.mar.23

[ref12] Cassese E. C. Farhart C. E. Miller J. M. (2020). Gender differences in COVID-19 conspiracy theory beliefs. Polit. Gender 16, 1009–1018. doi: 10.1017/S1743923X20000409

[ref13] Charlton E. (2014). Conspiracy theories and dissociative experiences: the role of personality and paranormal beliefs. Ph.D. thesis. London: Metropolitan University.

[ref14] Connolly J. M. Uscinski J. E. Klofstad C. A. West J. P. (2019). Communicating to the public in the era of conspiracy theory. Public Integr. 21, 469–476. doi: 10.1080/10999922.2019.1603045

[ref15] Correa T. Hinsley A. W. De Zuniga H. G. (2010). Who interacts on the web? The intersection of users’ personality and social media use. Comput. Hum. Behav. 26, 247–253. doi: 10.1016/j.chb.2009.09.003

[ref16] Dentith M. R. Orr M. (2017). Secrecy and conspiracy. Episteme 15, 433–450. doi: 10.1017/epi.2017.9

[ref17] Douglas K. M. Uscinski J. E. Sutton R. M. Cichocka A. Nefes T. Ang C. S.. (2019). Understanding conspiracy theories. Polit. Psychol. 40, 3–35. doi: 10.1111/pops.12568

[ref18] Dyrendal A. Hestad K. (2021). Trust in Crisis. Conspiracy mentality, lack of trust and religiosity predicted conspiracy beliefs about COVID-19 in a Norwegian sample. Approaching Religion 12, 98–114. doi: 10.30664/ar.107485

[ref19] Eberl J. M. Huber R. A. Greussing E. (2021). From populism to the “plandemic”: why populists believe in COVID-19 conspiracies. J. Elect. Public. Opin. Parties 31, 272–284. doi: 10.1080/17457289.2021.1924730

[ref20] Erceg N. Ružojčić M. Galić Z. (2020). Misbehaving in the Corona crisis: the role of anxiety and unfounded beliefs. Curr. Psychol. 41, 5621–5630. doi: 10.1007/s12144-020-01040-4, PMID: 34305363 PMC8280379

[ref21] Feldman G. (2021). Personal values and moral foundations: examining relations and joint prediction of moral variables. Soc. Psychol. Personal. Sci. 12, 676–686. doi: 10.1177/1948550620933434

[ref22] Freeman D. Waite F. Rosebrock L. Petit A. Causier C. East A.. (2020). Coronavirus conspiracy beliefs, mistrust, and compliance with government guidelines in England. Psychol. Med. 52, 251–263. doi: 10.1017/S0033291720001890, PMID: 32436485 PMC7264452

[ref23] Frenken M. Bilewicz M. Imhoff R. (2023). On the relation between religiosity and the endorsement of conspiracy theories: the role of political orientation. Polit. Psychol. 44, 139–156. doi: 10.1111/pops.12822

[ref24] Gallotti R. Valle F. Castaldo N. Sacco P. De Domenico M. (2020). Assessing the risks of ‘infodemics’ in response to COVID-19 epidemics. Nat. Hum. Behav. 4, 1285–1293. doi: 10.1038/s41562-020-00994-6, PMID: 33122812

[ref25] Georgiou N. Delfabbro P. Balzan R. (2020). COVID-19-related conspiracy beliefs and their relationship with perceived stress and pre-existing conspiracy beliefs. Personal. Individ. Differ. 166:110201. doi: 10.1016/j.paid.2020.110201, PMID: 32565592 PMC7296298

[ref26] Gligorić V. da Silva M. M. Eker S. van Hoek N. Nieuwenhuijzen E. Popova U.. (2021). The usual suspects: how psychological motives and thinking styles predict the endorsement of well-known and COVID-19 conspiracy beliefs. Appl. Cogn. Psychol. 35, 1171–1181. doi: 10.1002/acp.3844, PMID: 34177101 PMC8212084

[ref27] Goertzel T. (1994). Belief in conspiracy theories. Polit. Psychol. 15:731. doi: 10.2307/3791630

[ref28] Goreis A. Voracek M. (2019). A systematic review and meta-analysis of psychological research on conspiracy beliefs: field characteristics, measurement instruments, and associations with personality traits. Front. Psychol. 10:205. doi: 10.3389/fpsyg.2019.00205, PMID: 30853921 PMC6396711

[ref29] Gosling S. D. Rentfrow P. J. Swann W. B. (2003). A very brief measure of the big five personality domains. J. Res. Pers. 37, 504–528. doi: 10.1016/S0092-6566(03)00046-1

[ref30] Graham J. Haidt J. Koleva S. Motyl M. Iyer R. Wojcik S. P.. (2013). Moral foundations theory: the pragmatic validity of moral pluralism. Adv. Exp. Soc. Psychol. 47, 55–130.

[ref31] Graham J. Nosek B. A. Haidt J. Iyer R. Koleva S. Ditto P. H. (2011). Mapping the moral domain. J. Pers. Soc. Psychol. 101, 366–385. doi: 10.1037/a0021847, PMID: 21244182 PMC3116962

[ref32] Haidt J. Joseph C. (2004). Intuitive ethics: how innately prepared intuitions generate culturally variable virtues. Daedalus 133, 55–66. doi: 10.1162/0011526042365555

[ref33] Hartman T. K. Marshall M. Stocks T. V. McKay R. Bennett K. Butter S.. (2021). Different conspiracy theories have different psychological and social determinants: comparison of three theories about the origins of the COVID-19 virus in a representative sample of the UK population. Front. Polit. Sci. 3:642510. doi: 10.3389/fpos.2021.642510

[ref34] Heiss R. Gell S. Röthlingshöfer E. Zoller C. (2021). How threat perceptions relate to learning and conspiracy beliefs about COVID-19: evidence from a panel study. Personal. Individ. Differ. 175:110672. doi: 10.1016/j.paid.2021.110672, PMID: 33518866 PMC7825816

[ref35] Hettich N. Beutel M. E. Ernst M. Schliessler C. Kampling H. Kruse J.. (2022). Conspiracy endorsement and its associations with personality functioning, anxiety, loneliness, and sociodemographic characteristics during the COVID-19 pandemic in a representative sample of the German population. PLoS One 17:e0263301. doi: 10.1371/journal.pone.0263301, PMID: 35089987 PMC8797225

[ref36] Hollander B. A. (2018). Partisanship, individual differences, and news media exposure as predictors of conspiracy beliefs. Journal. Mass. Commun. Q. 95, 691–713. doi: 10.1177/1077699017728919

[ref37] Imhoff R. Lamberty P. (2020). A bioweapon or a hoax? The link between distinct conspiracy beliefs about the coronavirus disease (COVID-19) outbreak and pandemic behavior. Soc. Psychol. Personal. Sci. 11, 1110–1118. doi: 10.1177/1948550620934692 PMC7342934 38602949

[ref38] Infodemic. (2020). Available at: https://www.who.int/health-topics/infodemic (Accessed August 5, 2023).

[ref39] Iranian Students Poll Agency. (2021). Citizens' attitudes towards COVID-19 vaccination in Iran. Available at: http://www.ispa.ir/Default/Details/fa/2339 (Accessed August 5, 2023).

[ref40] Iyer R. Koleva S. Graham J. Ditto P. Haidt J. (2012). Understanding libertarian morality: the psychological dispositions of self-identified libertarians. PLoS One 7:e42366. doi: 10.1371/journal.pone.0042366, PMID: 22927928 PMC3424229

[ref41] Karić T. Međedović J. (2021). COVID-19 conspiracy beliefs and containment-related behaviour: the role of political trust. Personal. Individ. Differ. 175:110697. doi: 10.1016/j.paid.2021.110697, PMID: 33531725 PMC7843105

[ref42] Kim S. Kim S. (2021). Searching for general model of conspiracy theories and its implication for public health policy: analysis of the impacts of political, psychological, structural factors on conspiracy beliefs about the COVID-19 pandemic. Int. J. Environ. Res. Public Health 18:266. doi: 10.3390/ijerph18010266, PMID: 33396494 PMC7796329

[ref43] Lassen E. R. Hagen K. Kvale G. Eid J. Le Hellard S. Solem S. (2022). Personality traits and hardiness as risk-and protective factors for mental distress during the COVID-19 pandemic: a Norwegian two-wave study. BMC Psychiatry 22:610. doi: 10.1186/s12888-022-04237-y, PMID: 36109737 PMC9476397

[ref44] Leiser D. Duani N. Wagner-Egger P. (2017). The conspiratorial style in lay economic thinking. PLoS One 12:e0171238. doi: 10.2139/ssrn.2832354, PMID: 28257506 PMC5336227

[ref45] Leone L. Giacomantonio M. Lauriola M. (2019). Moral foundations, worldviews, moral absolutism and belief in conspiracy theories. Int. J. Psychol. 54, 197–204. doi: 10.1002/ijop.12459, PMID: 28875576

[ref46] Liekefett L. Christ O. Becker J. C. (2023). Can conspiracy beliefs be beneficial? Longitudinal linkages between conspiracy beliefs, anxiety, uncertainty aversion, and existential threat. Personal. Soc. Psychol. Bull. 49, 167–179. doi: 10.1177/01461672211060965, PMID: 34964375 PMC9896259

[ref47] Liu D. Campbell W. K. (2017). The big five personality traits, big two metatraits and social media: a meta-analysis. J. Res. Pers. 70, 229–240. doi: 10.1016/j.jrp.2017.08.004

[ref48] Marchlewska M. Cichocka A. Kossowska M. (2018). Addicted to answers: need for cognitive closure and the endorsement of conspiracy beliefs. Eur. J. Soc. Psychol. 48, 109–117. doi: 10.1002/ejsp.2308

[ref49] Marengo D. Sindermann C. Elhai J. D. Montag C. (2020). One social media company to rule them all: associations between use of facebook-owned social media platforms, sociodemographic characteristics, and the big five personality traits. Front. Psychol. 11:527189. doi: 10.3389/fpsyg.2020.00936, PMID: 32547442 PMC7273309

[ref50] McCrae R. R. Costa P. T. Jr. (1999). “A five-factor theory of personality” in Handbook of personality: theory and research. eds. Pervin L. A. John O. P. (United States: Guilford Press), 139–153.

[ref51] Nejat P. Hatami J. (2019). Psychometric properties of the Persian version of moral foundations questionnaire in three Iranian samples. J. Soc. Cogn. 8, 107–124. doi: 10.30473/sc.2019.40617.2204

[ref52] Nestik T. A. Deyneka O. S. (2020). Socio-psychological predictors of belief in conspiracy theories of the origin of COVID-19 and involvement in social media. Soc. Psychol. Soc. 11, 87–104. doi: 10.17759/sps.2020110407

[ref53] Nikčević A. V. Marino C. Kolubinski D. C. Leach D. Spada M. M. (2021). Modelling the contribution of the big five personality traits, health anxiety, and COVID-19 psychological distress to generalised anxiety and depressive symptoms during the COVID-19 pandemic. J. Affect. Disord. 279, 578–584. doi: 10.1016/j.jad.2020.10.053, PMID: 33152562 PMC7598311

[ref54] Nikpouraghdam M. Farahani A. J. Alishiri G. Heydari S. Ebrahimnia M. Samadinia H.. (2020). Epidemiological characteristics of coronavirus disease 2019 (COVID-19) patients in IRAN: a single center study. J. Clin. Virol. 127:104378. doi: 10.1016/j.jcv.2020.104378, PMID: 32353762 PMC7172806

[ref55] Olatunji O. S. Ayandele O. Ashirudeen D. Olaniru O. S. (2020). “Infodemic” in a pandemic: COVID-19 conspiracy theories in an african country. Soc. Health Behav. 3:152. doi: 10.4103/SHB.SHB_43_20

[ref56] Oleksy T. Wnuk A. Maison D. Łyś A. (2021). Content matters. Different predictors and social consequences of general and government -related conspiracy theories on COVID-19. Personal. Individ. Differ. 168:110289. doi: 10.1016/j.paid.2020.110289, PMID: 32834288 PMC7375289

[ref57] Orosz G. Krekó P. Paskuj B. Tóth-Király I. Bőthe B. Roland-Lévy C. (2016). Changing conspiracy beliefs through rationality and ridiculing. Front. Psychol. 7:1525. doi: 10.3389/fpsyg.2016.01525, PMID: 27790164 PMC5061726

[ref58] Pagliaro S. Sacchi S. Pacilli M. G. Brambilla M. Lionetti F. Bettache K.. (2021). Trust predicts COVID-19 prescribed and discretionary behavioral intentions in 23 countries. PLoS One 16:e0248334. doi: 10.1371/journal.pone.0248334, PMID: 33690672 PMC7946319

[ref59] Pérez-Mengual N. Aragonés-Barbera I. Moret-Tatay C. Moliner-Albero A. R. (2021). The relationship of fear of death between neuroticism and anxiety during the Covid-19 pandemic. Front. Psych. 12:648498. doi: 10.3389/fpsyt.2021.648498, PMID: 33959053 PMC8093560

[ref60] Pfeffer B. Goreis A. Reichmann A. Bauda I. Klinger D. Bock M. M.. (2022). Coping styles mediating the relationship between perceived chronic stress and conspiracy beliefs about COVID-19. Curr. Psychol. 1-9, 1–9. doi: 10.1007/s12144-022-03625-7, PMID: 35990199 PMC9381153

[ref61] Pivetti M. Di Battista S. Paleari F. G. Hakoköngäs E. (2021). Conspiracy beliefs and attitudes toward COVID-19 vaccinations: a conceptual replication study in Finland. J. Pac. Rim Psychol. 15:183449092110398. doi: 10.1177/18344909211039893

[ref62] Romer D. Jamieson K. H. (2020). Conspiracy theories as barriers to controlling the spread of COVID-19 in the U.S. Soc. Sci. Med. 263:113356. doi: 10.1016/j.socscimed.2020.113356, PMID: 32967786 PMC7502362

[ref63] Romero E. Villar P. Gómez-Fraguela J. A. López-Romero L. (2012). Measuring personality traits with ultra-short scales: a study of the ten item personality inventory (TIPI) in a Spanish sample. Personal. Individ. Differ. 53, 289–293. doi: 10.1016/j.paid.2012.03.035

[ref64] Sallam M. Dababseh D. Yaseen A. Al-Haidar A. Ababneh N. A. Bakri F. G.. (2020). Conspiracy beliefs are associated with lower knowledge and higher anxiety levels regarding COVID-19 among students at the University of Jordan. Int. J. Environ. Res. Public Health 17:4915. doi: 10.3390/ijerph17144915, PMID: 32650409 PMC7399915

[ref65] Shahsavari S. Holur P. Wang T. Tangherlini T. R. Roychowdhury V. (2020). Conspiracy in the time of corona: automatic detection of emerging COVID-19 conspiracy theories in social media and the news. J. Comput. Soc. Sci. 3, 279–317. doi: 10.1007/s42001-020-00086-5, PMID: 33134595 PMC7591696

[ref66] Šrol J. Čavojová V. Ballová Mikušková E. (2022). Finding someone to blame: the link between COVID-19 conspiracy beliefs, prejudice, support for violence, and other negative social outcomes. Front. Psychol. 12:6390. doi: 10.3389/fpsyg.2021.726076, PMID: 35095634 PMC8795973

[ref67] Stasielowicz L. (2022). Who believes in conspiracy theories? A meta-analysis on personality correlates. J. Res. Pers. 98:104229. doi: 10.1016/j.jrp.2022.104229

[ref68] Sternisko A. Cichocka A. Cislak A. Van Bavel J. J. (2023). National Narcissism predicts the belief in and the dissemination of conspiracy theories during the COVID-19 pandemic: evidence from 56 countries. Pers. Soc. Psychol. Bull. 49, 48–65. doi: 10.1177/01461672211054947, PMID: 34872399 PMC9684064

[ref69] Swami V. Chamorro-Premuzic T. Furnham A. (2010). Unanswered questions: a preliminary investigation of personality and individual difference predictors of 9/11 conspiracist beliefs. Appl. Cogn. Psychol. 24, 749–761. doi: 10.1002/acp.1583

[ref70] Swami V. Furnham A. (2012). Examining conspiracist beliefs about the disappearance of Amelia Earhart. J. Gen. Psychol. 139, 244–259. doi: 10.1080/00221309.2012.697932, PMID: 24837176

[ref71] Swami V. Pietschnig J. Tran U. S. Nader I. W. Stieger S. Voracek M. (2013). Lunar lies: the impact of informational framing and individual differences in shaping conspiracist beliefs about the moon landings. Appl. Cogn. Psychol. 27, 71–80. doi: 10.1002/acp.2873

[ref72] Tonković M. Dumančić F. Jelić M. Čorkalo Biruški D. (2021). Who believes in COVID-19 conspiracy theories in Croatia? Prevalence and predictors of conspiracy beliefs. Front. Psychol. 12:643568. doi: 10.3389/fpsyg.2021.643568, PMID: 34220613 PMC8249866

[ref73] Van Prooijen J. W. Douglas K. M. (2017). Conspiracy theories as part of history: the role of societal crisis situations. Mem. Stud. 10, 323–333. doi: 10.3389/fpsyt.2021.698147, PMID: 29081831 PMC5646574

[ref74] Van Prooijen J. W. Van Lange P. A. M. (2014). Power, politics, and paranoia: why people are suspicious of their leaders. Cambridge, UK: Cambridge University Press.

[ref75] Vranic A. Hromatko I. Tonković M. (2022). “I did my own research”: overconfidence, (dis) trust in science, and endorsement of conspiracy theories. Front. Psychol. 13:931865. doi: 10.3389/fpsyg.2022.931865, PMID: 35910977 PMC9330604

[ref76] Wang J. L. Jackson L. A. Zhang D. J. Su Z. Q. (2012). The relationships among the big five personality factors, self-esteem, narcissism, and sensation-seeking to Chinese university students’ uses of social networking sites (SNSs). Comput. Hum. Behav. 28, 2313–2319. doi: 10.1016/j.chb.2012.07.001

[ref77] World Health Organization. (2020). WHO Director-General’s opening remarks at the media briefing on COVID-19-11 March 2020. Available at: https://www.who.int/director-general/speeches/detail/who-director-general-s-opening-remarks-at-the-media-briefing-on-covid-19---11-march-2020 (Accessed August 5, 2023).

[ref78] Yilmaz O. Saribay S. A. (2019). Moral foundations explain unique variance in political ideology beyond resistance to change and opposition to equality. Group Process. Intergroup Relat. 22, 1124–1138. doi: 10.1177/1368430218781012

[ref79] Zacher H. Rudolph C. W. (2021). Big five traits as predictors of perceived stressfulness of the COVID-19 pandemic. Personal. Individ. Differ. 175:110694. doi: 10.1016/j.paid.2021.110694, PMID: 33531723 PMC7843115

[ref80] Zonis M. Joseph C. M. (1994). Conspiracy thinking in the Middle East. Polit. Psychol. 15:443. doi: 10.2307/3791566

