# Supplementary material for: Moral foundations are better predictors of belief in COVID-19 conspiracy theories than the Big Five personality traits
Source: Front Psychol. 2023 Aug 24;14:1201695. doi: 10.3389/fpsyg.2023.1201695 (PMC10484408; doi:10.3389/fpsyg.2023.1201695)
Supplement: Supplementary file 1 [file Table_1.DOCX]

Supplementary Material

Moral Foundations are Better Predictors of Belief in COVID-19 Conspiracy Theories than the Big Five Personality Traits

Pegah Nejat*, Ali Heirani-Tabas, Mohammad Mahdi Nazarpour

*** Correspondence:** Pegah Nejat: p_nejat@sbu.ac.ir

# Supplementary - Belief in Covid-19 conspiracy theories items

**Lockdown (Three items)**

1- Lockdown is a way to terrify, isolate, and demoralise a society as a whole in order to reshape society to fit specific interests.

2- The intention of lockdown is to force people to rely on big corporations rather than local businesses.

3- Lockdown is a plot by environmental activists to control the rest of us.

**Vaccination (Three items)**

4- The coronavirus vaccine contains microchips to control the people.

5- The vaccine will be used to carry out mass sterilisation.

6- The coronavirus is bait to scare the whole globe into accepting a vaccine that will introduce the ‘real’ deadly virus.

**Origin of virus (21 Items, four reverse-scored)**

7- Big Pharma created coronavirus to profit from the vaccines.

8- Coronavirus was created to force everyone to get vaccinated.

9- Coronavirus is a bioweapon developed by China to destroy the West.

10- The virus is a biological weapon manufactured by the United States.

11- The spread of the virus is a deliberate attempt to reduce the size of the global population.

12- The spread of the virus is a deliberate attempt by governments to gain political control.

13- The spread of the virus is a deliberate attempt by a group of powerful people to make money.

14- The spread of the virus is a deliberate attempt by a group of powerful people to gain control.

15- The spread of the virus is a deliberate attempt by one nation to destabilise another.

16- The spread of the virus is a deliberate attempt by global companies to take control.

17 - The spread of the virus is a deliberate attempt by activists to stop climate change.

18- The UN and WHO have manufactured the virus to take global control.

19- Jews have created the virus to collapse the economy for financial gain.

20- The elite have created the virus in order to establish a one-world government.

21- Bill Gates has created the virus in order to reduce the world population.

22- The coronavirus is a natural infectious disease that has spread worldwide and caused a pandemic (R).

23- The virus is naturally occurring (R).

24- The virus has been transmitted to us from wild animals (R).

25- I think it’s nonsense that the virus was created in a laboratory (R).

26- Coronavirus is a plot by globalists to destroy religion by banning gatherings.

27- Coronavirus is an alien weapon to destroy humanity.

**Exploiting Covid for other purposes (Three items)**

28- The virus is a front to implement measures to destroy our privacy.

29- Coronavirus is a news only to divert attention from more important things.

30- Antibody testing is a plot to harvest our DNA.

**News accuracy (Four Items, two reverse-scored)**

31- The mainstream media is deliberately feeding us misinformation about the virus and lockdown.

32- Social distancing reduces the likelihood of spreading the virus from person to person (R).

33- We should believe experts when they say that the virus is dangerous (R).

34- Coronavirus cannot be passed from person to person, you can only get it if someone deliberately infects you with it (e.g., being injected or poisoned).
